# Innovation in neglected tropical disease drug discovery and development

**DOI:** 10.1186/s40249-018-0444-1

**Published:** 2018-06-18

**Authors:** Hong-Bo Weng, Hai-Xia Chen, Ming-Wei Wang

**Affiliations:** 1 School of Pharmacy, Fudan University, 826 Zhangheng Road, Pudong New District, Shanghai, 201203 China; 2 The National Center for Drug Screening and the CAS Key Laboratory of Receptor Research, Shanghai Institute of Materia Medica, Chinese Academy of Sciences (CAS), 189 Guoshoujing Road, Pudong New District, Shanghai, 201203 China; 3 School of Life Science and Technology, ShanghaiTech University, 393 Middle Huaxia Road, Pudong New District, Shanghai, 201210 China

**Keywords:** Innovation, Collaboration, Open-source, Neglected tropical diseases

## Abstract

**Background:**

Neglected tropical diseases (NTDs) are closely related to poverty and affect over a billion people in developing countries. The unmet treatment needs cause high mortality and disability thereby imposing a huge burden with severe social and economic consequences. Although coordinated by the World Health Organization, various philanthropic organizations, national governments and the pharmaceutical industry have been making efforts in improving the situation, the control of NTDs is still inadequate and extremely difficult today. The lack of safe, effective and affordable medicines is a key contributing factor. This paper reviews the recent advances and some of the challenges that we are facing in the fight against NTDs.

**Main body:**

In recent years, a number of innovations have demonstrated propensity to promote drug discovery and development for NTDs. Implementation of multilateral collaborations leads to continued efforts and plays a crucial role in drug discovery. Proactive approaches and advanced technologies are urgently needed in drug innovation for NTDs. However, the control and elimination of NTDs remain a formidable task as it requires persistent international cooperation to make sustainable progresses for a long period of time. Some currently employed strategies were proposed and verified to be successful, which involve both mechanisms of ‘Push’ which aims at cutting the cost of research and development for industry and ‘Pull’ which aims at increasing market attractiveness. Coupled to this effort should be the exercise of shared responsibility globally to reduce risks, overcome obstacles and maximize benefits. Since NTDs are closely associated with poverty, it is absolutely essential that the stakeholders take concerted and long-term measures to meet multifaceted challenges by alleviating extreme poverty, strengthening social intervention, adapting climate changes, providing effective monitoring and ensuring timely delivery.

**Conclusions:**

The ongoing endeavor at the global scale will ultimately benefit the patients, the countries they are living and, hopefully, the manufacturers who provide new preventive, diagnostic and therapeutic products.

**Electronic supplementary material:**

The online version of this article (10.1186/s40249-018-0444-1) contains supplementary material, which is available to authorized users.

## Multilingual abstracts

Please see Additional file 1 for translations of the abstract into the five official working languages of the United Nations.

## Background

Neglected tropical diseases (NTDs) are rife among the world’s poorest populations living in tropical and sub-tropical developing countries with limited resources, often reflected by deficient medical infrastructure and sub-standard sanitation. Endemic populations also tend to live in close proximity to domestic animals and livestock, which exacerbates the prevalence and propagation of NTDs. The World Health Organization (WHO) indicates that more than one billion people are afflicted with one or more NTDs in 149 countries [1], and NTDs cause over 35,000 deaths per day worldwide [2]. In terms of long-term disability, illness and death, NTDs impose a heavy burden with severe social and economic consequences in the developing world. The lack of attention paid to these diseases is out of all proportion to their global importance. NTDs have been largely ignored by drug manufacturers and public policy makers for decades. As a result, the control of NTDs is still inadequate and extremely difficult today.

Although some organizations and infectious disease experts define NTDs differently, WHO has specifically identified 17 priority NTDs caused by four different classes of pathogens in 2011: viruses, bacteria, protozoa, and helminths. These diseases are Chagas disease, human African trypanosomiasis, leishmaniasis, Buruli ulcer, leprosy, trachoma, yaws, cysticercosis/taeniasis, dracunculiasis, echinococcosis, food borne trematodiases, lymphatic filariasis, onchocerciasis, schistosomiasis, soil-transmitted helminthiasis, dengue and chikungunya, as well as rabies [3]. Mycetoma, chromoblastomycosis and other deep mycoses, scabies and other ectoparasites, as well as snakebite envenoming were added to the list in 2017. In fact, WHO has established the goals of eliminating 5 diseases (leprosy, sleeping sickness, blinding trachoma, guinea worm disease and lymphatic filariasis) and controlling another 5 (schistosomiasis, helminthiases, visceral leishmaniasis, onchocerciasis and Chagas disease) by 2020 [4]. However, besides the inadequacy of sanitation conditions, the lack of safe, effective and affordable medicines is also identified as a key contributing factor that may hinder the achievement of these targets [5].

Historically, NTDs have been ignored by the pharmaceutical industry and public health coverage in general. Most people infected with NTDs are confronted with poor sanitary conditions and have inadequate nutrition and healthcare. They are unable to pay for treatment, even if available [6]. For the pharmaceutical industry, drugs to mitigate NTDs do not bring sufficient economic returns such that little incentive exists to spur its commercial interest for research and development (R&D). As a result, very few new therapeutic agents have been launched for NTDs in recent years.

## Insufficient drug innovation for NTDs

Drugs, vaccines, diagnostics and vector control products are important tools for the prevention and treatment of NTDs. Compared to other diseases, a very small number of new therapeutics for NTDs implies a persistent gap in innovation. In the period between 1975 and 1999, 1393 new drugs were approved but only 13 (0.93%) of them were for NTDs [7]. In the subsequent 10 years, the situation was not significantly improved: of the 850 new therapeutic products registered between 2000 and 2011, only 5 (0.59%) were indicated for NTDs, and all of which were cataloged for new indication or formulation of existing drugs; none were new chemical entities [8]. From 2000 to 2014, 66 novel products entered phase I clinical trials intended to prevent or treat NTDs. It accounts for 1.65% of all 4006 phase I trials [9].

It is known that drug discovery and development (DDD) is very costly with inherent risk of failure. The drug innovation outcomes for a particular disease reflect the investment in R&D. Funding limitation restricted the innovation efforts in this area. US$307 million per million disability-adjusted life years (DALYs ) is used worldwide on non-infectious respiratory diseases, whereas only US$3 million per million DALYs was for NTDs in 1999 [7]. In 2010, the US$2.4 billion investment in NTDs accounted for only 1% of overall healthcare-related research expenditure [10]. Fortunately, funding situation for NTDs increased notably to US$3.045 billion in 2011 [11]. However, the increase in investment is still a small amount compared with expenditures on non-neglected disease R&D. Nevertheless, the development of new medical products against NTDs has been promoted. In the period between 2000 and 2013, there were three new approvals of NTD products, and five products targeting NTDs were in phase III clinical trials including one for Dengue fever, three for onchocerciasis/schistosomiasis and one for all three diseases (leishmaniasis, Chagas disease and African sleeping sickness) [12].

## Collaborative efforts in drug discovery

It should be said that recent international efforts in balancing the investment between neglected and other diseases have begun to reshape the landscape. In January 2012, nine pharmaceutical company top executives, the chief of WHO, Mr. Bill Gates and several political leaders from different countries signed the London Declaration on Neglected Tropical Diseases, a roadmap guiding the implementation of policies and strategies on NTDs [13]. It emphasizes the importance of increasing funding to improve implementation and pledges to control or eliminate 10 of the 17 targeted NTDs by 2020 [14]. According to the first annual report, the projected funding gap is approximately US$300 million per year [15]. The third progress report indicated that pharmaceutical companies had pledged drugs valued at US$17.8 billion for the 10 diseases through 2020 [16]. During the Global Partners Meeting on April 19, 2017, governments, partners, philanthropists and industry representatives pledged a cumulative total of $800 million over the next 5 to 7 years to accelerate the elimination and eradication of NTDs [17]. Recently, WHO has welcomed the launch of a US$100 million fund to facilitate the elimination of two devastating infectious NTDs: onchocerciasis (river blindness) and lymphatic filariasis (elephantiasis). The Crown Prince of Abu Dhabi and Bill & Melinda Gates Foundation have each committed US$20 million while the remaining US$60 million will be raised by working with other donors and foundations [17]. At the Global Partners Meeting 2017, WHO reported remarkable achievements in tackling NTDs since 2007 [17].

The product development partnership (PDP) model now plays a crucial role in drug innovation, which usually involves research institutions, pharmaceutical companies, government agencies and international organizations, such as the Drugs for Neglected Disease Initiative (DNDi) [18]. Their contributions to NTD treatment have been well-documented [19–21], indicating that most cooperation took place through PDPs via fostering a high-performance collaborative approach to drug discovery for NTDs [22]. As a global vehicle of scientific collaboration, the Special Programme for Research and Training in Tropical Diseases (TDR) under WHO has assisted in the establishment of PDPs and supported research and control of NTDs for 30 years since its inception [23, 24]. It worked with industry partners in the development of new products, such as eflornithine for African trypanosomiasis, praziquantel for schistosomiasis, and various drug combination and formulation for malaria [25]. Although remaining moderately successful, its influence has waned in the past decade because of the dynamic and changing landscape of global health, such as priority resetting and the emergence of public–private partnerships (PPPs) involved in product development. TDR was implicated that it neither well managed its partnership with other donor agencies and clearly defined respective functions and tasks, nor built sufficient linkages and mutual agreements for collaboration. Its strengths and successes require vigorous search for better and more effective means to partner with others [25].

## Efficient approaches for drug innovation

Although recent increases in funding have accelerated the development of novel treatments for NTDs, some fundamental barriers still exist. Because the parasitic pathogens involved have rather complex biology and requirements for vectors for their development and transmission, conventional drug discovery approaches are encountering incredible challenges. Many basic research findings have yet to find their way to drug discovery programs and the lack of screening platforms, appropriate tools, suitable assays and validated targets has further hampered the process.

Practically, both traditional target-based and phenotype-based screening assays are often employed in drug discovery, particularly for NTDs [26, 27]. However, for anti-leishmanial agents, target-based screenings showed some drawbacks. Since the intracellular amastigote situates inside of the macrophage parasitophorous vacuole, putative hits should have high permeability to pass across several membranes in addition to possessing an excellent stability in an acidic environment. Moreover, they should not interact with xenobiotic-metabolizing enzymes as substrates both in the host and the parasite [28]. For human African trypanosomiasis treatment, it was found that targeting N-myristoyltransferase in *Trypanosoma brucei* was successful while inhibiting pteridine reductase 1 (PTR1) was a failure [27]. Clearly, target-based screening can be successful, but requires careful selection of targets. For NTDs with few validated drug targets, a high attrition rate exists in target-based drug discovery. This approach has been impeded by the lack of genetic tools to validate drug targets in these parasites. However, phenotypic screening can reflect all the targets and biological pathways as whole organism being exposed to the chemical entities in testing [29]. The approach to identification of molecular targets following a phenotypic screen has been proven most successful [26]. Thus, running an upfront phenotypic assay in primary screening is thought to be a viable alternative to discover hits as chemical probes for eventual target identification. Taken together, a balanced portfolio that includes target-based approaches with careful target selection and phenotype-based methods might be the best strategy in this regard.

Phenotypic screening approaches have been developed in both academic and pharma settings. It is recognized that a well-understanding of host-parasite interaction and the disease itself is essential to the design of better and more predictive phenotypic assays, considering unique characteristics and complexity of the parasite, its life cycle, and its interaction with host cells [30]. For example, protozoan parasites have devised sophisticated mechanisms to elude the defense response of the host. Some are able to live within immune cells by hijacking important components of the host defense [31].

It has been debated that the chemical diversity of molecules routinely used for screening is more suited to host targets. Given the low hit rates for pathogens such as *Leishmania* [29] and *Schistosoma* [32], looking for alternative sources of high-quality chemical compounds is necessary. Large-scale screening of natural products and extracts might be a feasible way to inspect more diversity in the chemical space for NTDs [33].

## Technology advancement in drug discovery

Automated microscopy has taken the significant advances in drug discovery for NTDs [34–39]. For example, it increased screening throughput by replacing laborious and subjective manual microscopic observation in helminth studies [40]. High content imaging was also applied to assay schistosomes, while algorithm methods were developed to identify morphological injury and changes in motility of schistosomula (the larval stage of the parasite) in 384-well microtiter plates. This high content screening (HCS) technology was tested and verified against either 10,041 compounds with demonstrated consistency of visual inspection [41] or helminths utilizing an automated motion-based platform [41]. Meanwhile, worm-based assays have both been evaluated with *Brugia malayi* (the pathogen of lymphatic filariasis) and schistosomes, indicating that this method is applicable to a variety of macroparasites [42]. For some protozoan parasites, such as *Trypanosoma brucei* (the pathogen of African sleeping sickness), high-throughput screening (HTS) of compound libraries were possible without the need for high content imaging [43, 44]. However, for other protozoa such as *Trypanosoma cruzi* (the pathogen of Chagas disease) [45, 46] and *Leishmania* species [34, 39], HCS provides the opportunity of improving and building on previous screens through its ability of analyzing compounds against more clinically relevant stages of parasite life cycle.

Since various pathogens involved in NTDs have a complex life cycle, drug sensitivity could change with different life cycle stages. This has recently been demonstrated with bioassays targeting *Leishmania*. Only 4% of the hits identified by a promastigote (the stage in the insect) screen were confirmed by intracellular amastigote (the stage in the human) assessment [34]. For anti-trypanosomatid therapeutics, it is prudent to use an appropriate combination of assays. For example, an HTS assay is generally required to identify initial hits with a broad structural diversity, followed by confirmation studies with more physiologically relevant, but lower throughput assays. Cellular assays are representatives of certain *in vivo* situation and suitable to preliminary evaluation of pharmacokinetic and/or pharmacodynamic profiles [47].

Inappropriate screening technologies could result in false negative or false positive data. Therefore, it is critical to select appropriate technologies or methods to study these parasites, with the willingness of applying new methodologies [35]. For example, metabolomics studies were carried out to identify pathways and targets for hits found by phenotypic screens [48]. Yeast-based method was shown to be amenable to HTS of parasitic target in cellular milieu [49]. Bioluminescent live-imaging facilitated the observation of infection caused by genetically modified parasites and drug effect in the process [50]. Fragment- or structure-based screening was demonstrated to be a novel way to optimize DDD for NTDs [51–55].

## Successful strategy for drug innovation

**Fig. 1:**
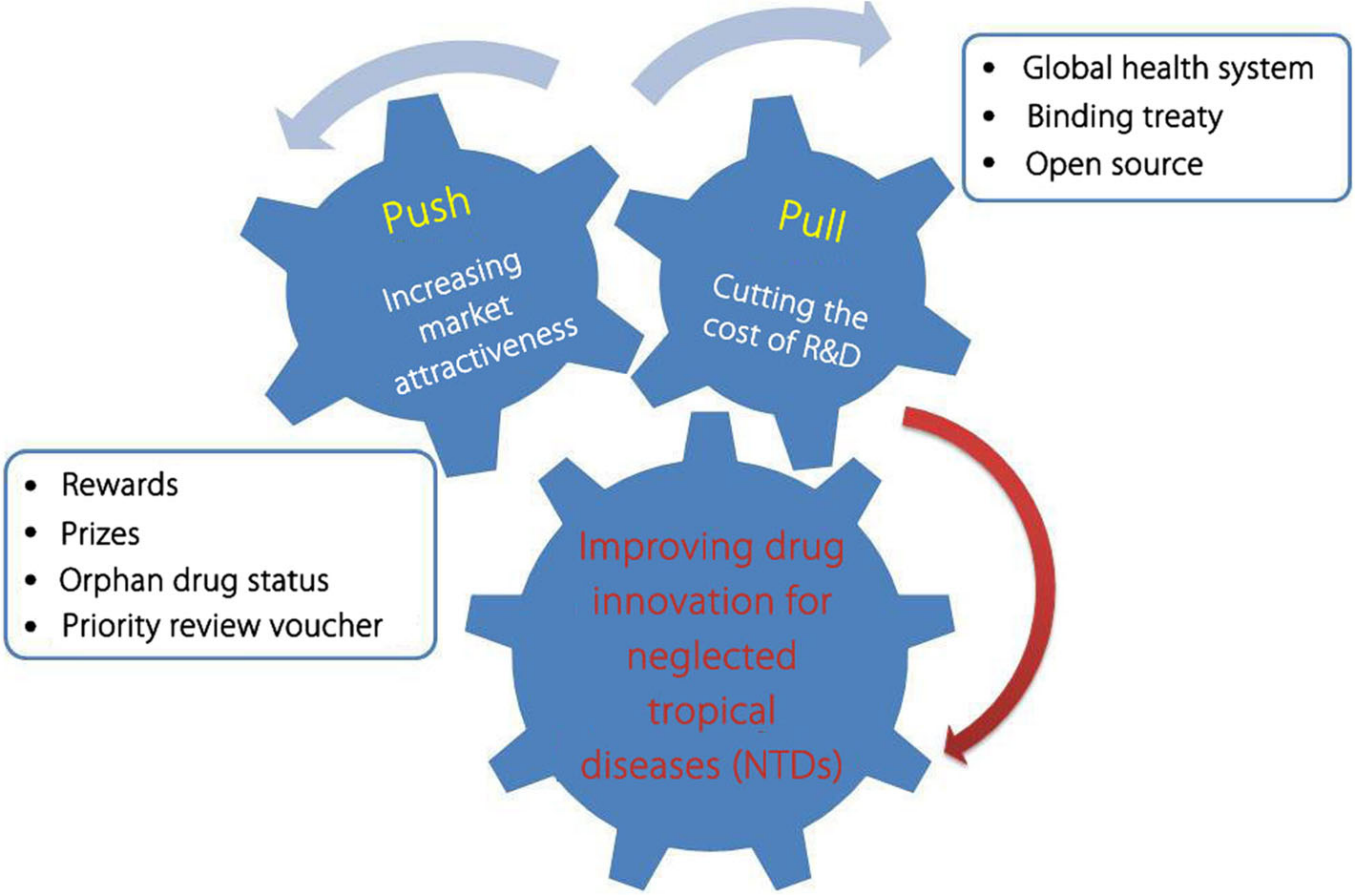
Strategic approaches to neglected tropical disease drug discovery and development

To address the lack of NTD drug innovation and financing, both mechanisms of ‘Push’ which aims at cutting the cost of R&D for industry and ‘Pull’ which aims at increasing market attractiveness were proposed and verified to be successful [56, 57]. Some currently employed strategies in the fight against NTDs are highlighted in Fig. 1.

An internationally binding treaty focusing on mobilizing global resources towards healthcare priorities and promoting NTD-related DDD was suggested [58–60]. This proposal has gained support from many stakeholders such as PPPs, pharmaceutical companies, academic institutions, *etc*. Based on the WHO treaty instrument, it stipulates that national governments should fix their legal obligation to provide a minimum investment for ensuring sustainability [58]. The establishment of the treaty would lay the foundation for a new global health system thereby facilitating innovative drug development for NTDs. Such a global health system with secured funding mechanisms is crucial to control and eliminate NTDs. Under the leadership of WHO, the global fund is operated by a multi-stakeholder partnership and has become a principal institution dedicated to NTDs in the world [61]. This seems a logical model to manage the complexity of NTDs which require low-cost drugs available as generics, sustainable funding mechanisms, harmonization of stakeholder/donor activities and healthcare systems emphasizing treatment delivery. Indeed, many partnerships associated with NTDs have thus engaged the private sector in drug discovery and medicine donations [61] by use of the operational expertise and governance structure of the global fund. In 2013, the Global Health Innovative Technology Fund (GHIT Fund), a new PPP between the government of Japan, five Japanese pharmaceutical companies (Astellas Pharma, Daiichi Sankyo, Shionogi & Co., Eisai and Takeda) and an international philanthropic foundation (Bill & Melinda Gates Foundation), was launched. Its initial aim is to expedite the development of novel technologies and drugs against HIV/AIDS, malaria, tuberculosis and NTDs designated by WHO. GHIT Fund has committed a potential 5-year investment of more than $100 million [62].

Rewards are given for successful drug development as a pull mechanism to promote innovation [57]. Rewards can be designed in a variety of ways: to be specifically focused on one medical product, for example, to be more like grants; or open to a wide variety of medical products distributed according to public health benefits [63]. Criteria for eligibility can be set to help achieve development goals: for instance, medicines might be required to have a viable mechanism for delivery in resource-poor areas in order to qualify. Rewards may also include fixed awards, milestone requirement to space out payments and prizes based on outcome measures such as impact on DALYs [64]. The amount of reward should be predicated on efficacy over and above the current state of the art, rather than above the placebo, in order to discourage the drugs that have lower benefits for public health although they possess less R&D risks [63]. For prizes, the need to separate the drug price from R&D incentives is critical to ensure accessibility [57]. However, there is an inherent difficulty in specific calculation of the prize value. It was suggested that the more neglected a disease and the greater the prize: e.g., for diseases found either overwhelmingly or exclusively in developing countries, high prizes should be given to encourage innovation, because there is often no market and therefore no incentive for research in these regions [65]. To reduce risk of potential overpayment for unfinished research, payment terms must be carefully drafted to provide incentive for participation. Usually, paying premiums is a way to make up for the investment failures [64].

Open source is a new strategy that may foster innovations in preventive, diagnostic and therapeutic products for NTDs. Limited market size and inadequate interaction between academia and industry are the two obstacles that decelerate the development of new drugs. However, open source focuses on encouraging collaboration and resource-sharing between academic institutions and pharmaceutical companies where they could access talents, resources, tools and technologies from each other [57]. The Human Genome Project (HGP) was the first example in the open source model. As a tool and resource platform, HGP provides human genome sequencing to researchers worldwide, which led to some well-documented advances in drug discovery [66]. The unequivocal success of this model in software area remarkably fills the niche neglected by the private sector [67]. Another example of the open source model relates to the Tres Cantos Open Lab Foundation (TCOLF) which allows academics to use GlaxoSmithKline’s facilities and knowhow in Spain. It has activated an unprecedented degree of scientific exchanges leading to numerous research grants and follow-up engagement [68]. In addition, open source collaboration promotes more academic reach-out, for example, more peer-reviewed journals transform into an open-access format, which is beneficial for disseminating research outcomes more broadly [66, 67, 69, 70].

An open source model for drug innovation can only be effective when information exchange and data sharing mechanism cover both discovery and development stages [71]. Clearly, this requires very complicated process of bilateral and/or multilateral confidentiality commitments and arrangements. At present, the open source model appears working well for NTDs, as most of the stakeholders involved are nonprofit or philanthropic organizations collaborating with pharmaceutical or biotech companies [71]. The Pathogen Box from Medicines for Malaria Venture (MMV) has been proven to be a successful open-access for NTDs drug research [72–74].

The Pathogen Box comprised of approximately 400 compounds active against NTDs, such as Chagas’, human African trypanosomiasis and schistosomiasis. Researchers around the world can freely request a Pathogen Box without charge, which will promotes the establishment of an open and collaborative forum of DDD for NTDs. Recently, the three kinetoplastid chemical boxes offered by GlaxoSmithKline has become an open resource for future lead discovery programs [75]. They were assembled with approximately 200 compounds each and are freely available to academic researchers. Free flow of information is one of the goals of this model: it would not only facilitate innovation but also encourage competition. The latter is instrumental to the production of less expensive and more accessible medicines: two major targets in combating NTDs.

Pharmaceutical companies sometimes engage in product development for rare diseases, *i.e*., orphan drug indications. However, financial support and market incentives are necessary to do so [76]. This approach has also been suggested to incentivize NTD drug innovation [76, 77], including but not limited to fast-track regulatory processes, clinical trials assistance, exemption from registration fees, tax credits and other financial constraints [76]. Nevertheless, in developed countries, orphan drug laws are criticized because they prioritize private sector profits in the form of market exclusivity with inadequate guarantees for patient accessibility or affordability [76, 78]. In contrast, in resource-stretched countries, the mechanism may bring a risk that administration could not fully control the price concessions of drugs [77].

To encourage drug discovery for NTDs, Food and Drug Administration (FDA) in the U.S.A. created a priority review voucher (PRV) program in 2007 [79]. It allows pharmaceutical companies to fast-track review for a new drug application (NDA). Although the voucher does not guarantee approval by the FDA, it does guarantee that a drug will be reviewed and have a decision rendered within six months. In addition, it can be used by the recipient manufacturer for another development program, or sold to another company for use. The scheme is an important incentive for pharmaceutical companies to conduct research on medicines for NTDs that might otherwise not be profitable to develop. To date, there have been four NDAs for rare tropical diseases that received the support of this program [80]. However, criticisms on the effectiveness of the PRV program also surfaced [81]. The incentive of PRV is considered to be “late-stage” that only pushes products to the market rather than initiating early-stage R&D. Some of the products approved are not necessarily “novel” in terms of innovation. Another significant concern is that there is no drug affordability or accessibility requirement after garnering a PRV, e.g., pricing it appropriately or registering it in the countries where it is most needed [82].

## Facing challenges in drug discovery

Despite increased funding and advocacy prompted technological advances, DDD for NTDs is still facing many challenges. First, geographic, environmental and cultural aspects are also important contributing factors to predispose individuals to NTDs [83–87]. It is known that a numbers of NTDs are zoonotic and most are vector borne. Therefore, control strategies based only on treating the infected human population are unlikely to be successful. On the other hand, in consideration of financial returns, many pharmaceutical companies prefer to develop new therapeutic agents against NTDs by repurposing existing drugs for other indications in order to reduce R&D costs [88, 89]. This makes discovery of new chemical entities for NTDs much less attractive. Nevertheless, repurposing of existing clinical candidates or drugs is undergoing a resurgence of interest, which is attributed to the status of slow and uninspiring drug discovery for NTDs [90–92]. It is suggested that repurposing of existing drugs can satisfy an urgent requirement for a safe, rapid and cost-effective treatment of NTDs. Furthermore, widespread drug resistance is also a major challenge in current clinical therapy. For instance, current anti-trypanosomal agents, such as pentamidine, suramin, melarsoprol and eflornithine, are beset by the emergence of drug resistance. Praziquantel was reported to have reduced efficacy in some schistosome strains raising worries of development of resistance. For almost seven decades pentavalent anti-monials constituted the standard anti-leishmanial treatment worldwide, however, their clinical value was jeopardized in the past 10 to 20 years due to the development resistance to these agents, e.g., in North Bihar in India [73, 74]. Thus, close medical supervision is strongly recommended to ensure reasonable use of drugs. For countries where NTDs prevail, resource-poor settings may be another obstacle to relieve drug resistance by performing complicated drug administration procedures.

## Conclusions

NTDs are complex and involve a number of factors associated with geographic, environmental, economic and social issues. They have become a global public health concern and require multidisciplinary intervention to contain the prevalence in many parts of the world. It is obvious that DDD in this very specialized but less- or non-profitable area has encountered tremendous difficulties and the challenges we are facing call for collective efforts by the international community at large. Notable initiatives described in this review, such as novel financing models, incentive schemes, PDPs, policy advocacy and innovation in technology, nowadays play important roles in accelerating the control and eventual elimination of NTDs.

To expand such a positive development, new leads must be continuously generated via the open source mechanism. One successful example relates to the establishment of the Chinese National Compound Library (Shanghai) in 2008 via a PPP, consisting of the National Center for Drug Screening (China), Shanghai Institute of Materia Medica, Chinese Academy of Sciences, WHO and Novo Nordisk A/S (Denmark) [93]. This resource-sharing arrangement has not only provided unprecedented opportunities to scientists around the globe who are interested in accessing large quantities of small-molecule compounds, but also trained dozens of young scholars from both developing and developed countries in conducting HTS campaigns against molecular targets associated with several NTDs [32, 94, 95]. It is expected that by taking advantage of this large and diverse collection of compounds (currently possessing more than 2.2 million chemical entities), the efficiency of NTD-related drug innovation will be greatly improved.

Achieving the 2020 goals of WHO by eliminating 5 and controlling another 5 NTDs [4] is a shared responsibility of the international community. Therefore, collaborative innovation via the open source mechanism is an indispensible way to coordinate various stakeholders towards a common target: discovering and developing new drugs to combat the spread of NTDs. In the existing PDP models, PPP shows its promise in term of result-oriented execution. The bond may be further strengthened if the concern of the private sector (*i.e.*, return on investment) could be taken into consideration through granting of orphan drug status or advance market commitment (AMC). Another exhibit of PPP is characterized by distribution of financial returns to all partners in the event that their joint development project is successful. This may ensure a self-funding scheme that provides sustainability in supporting innovation.

In summary, the control and elimination of NTDs remains a formidable task. One of the hurdles is the lack of more effective and safer treatment. As a result, drug innovation for NTDs is not only urgently needed but also requires both conventional and advanced technologies. Coupled to this effort should be the exercise of shared responsibility globally to reduce risks, overcome obstacles and maximize benefits. Since NTDs are closely associated with poverty, it is absolutely essential that the stakeholders take asserted and long-term measures to meet multifaceted challenges by alleviating extreme poverty, strengthening social intervention, providing effective monitoring and ensuring timely delivery.

## Additional file

Additional file 1: Multilingual abstracts in the five official working languages of the United Nations. (PDF 234 kb)

## Abbreviations

AMC: Advance market commitment
DALYs: Disability-adjusted life years
DDD: Drug discovery and development
DNDi: Drugs for Neglected Disease Initiative
FDA: Food and Drug Administration
GHIT: Global Health Innovative Technology
HCS: High content screening
HGP: Human Genome Project
HTS: High-throughput screening
MMV: Medicines for Malaria Venture
NDA: New drug application
NTDs: Neglected tropical diseases
PDP: Product development partnership
PPPs: Public–private partnerships
PRV: Priority review voucher
PTR1: Pteridine reductase 1
TCOLF: Tres Cantos Open Laboratory Foundation
TDR: Special Programme for Research and Training in Tropical Diseases
WHO: World Health Organization
