# Supplementary material for: Innovation in neglected tropical disease drug discovery and development
Source: Infect Dis Poverty. 2018 Jun 18;7:67. doi: 10.1186/s40249-018-0444-1 (PMC6022351; doi:10.1186/s40249-018-0444-1)
Supplement: Supplementary file 1 — Multilingual abstracts in the five official working languages of the United Nations. (PDF 234 kb) [file 40249_2018_444_MOESM1_ESM.pdf]

Translation of the abstract into the five official working languages of the United Nations

الابتكار في اكتشاف العقاقير للأمراض المدارية المهملة وتطويرها

قدمه: هونغبو وينغ، وهايكسيا شين، ومينغ وي وانغ

الملخص
ترتبطُ الأمراض المدارية المهملة ارتباطًا وثيقًا بالفقر وتؤثر على ما يزيد عن مليار شخص في الدول النامية. تتسبب عدم تلبية احتياجات العلاج بارتفاع معدل الوفيات وبالعجز. وبالتالي، فرض عبئًا ثقيلًا مع ترتب عواقب اجتماعية واقتصادية وخيمة. فعلى الرغم من تولي منظمة الصحة العالمية تنسيق هذه الجهود، إضافةً إلى العديد من المنظمات الخيرية، والحكومات الوطنية وصناعة الأدوية ما زالت تبذل جهدها في تحسين الوضع. ومع ذلك ما تزال السيطرة على الأمراض المدارية المهملة غير كافية وفي غاية الصعوبة. وهناك عامل مساهم رئيسي وهو شح وجود الأدوية الآمنة والفاعلة وبأسعار معقولة. تستعرض هذه الورقة البحثية التطورات الحديثة وبعض التحديات التي نواجهها في مكافحة الأمراض المدارية المهملة.
في السنوات الأخيرة، أظهرت العديد من الابتكارات ميلًا لتعزيز اكتشاف العقاقير وتطويرها للأمراض المدارية المهملة. يؤدي تنفيذ التعاون متعدد الأطراف إلى مواصلة الجهود وله دور حاسم في اكتشاف العقاقير. هناك حاجة ملحة لوجود النُهج استباقية والتقنية المتقدمة في ابتكار العقاقير للأمراض المدارية المهملة. ومع ذلك، تبقى السيطرة على هذه الأمراض وإبادتها مهمة صعبة، إذ تتطلب تعاونًا عالميًا مستمرًا للوصول إلى تقدم مستدام لبرهة طويلة من الزمن. تم اقتراح بعض آليات العمل الحالية وتمت المصادقة على نجاحها، تتضمن آليتان، آلية "الدفع" والتي تهدف الى تقليل تكاليف بحث الصناعة وتطويرها، وآلية "السحب" والتي تهدف إلى زيادة جاذبية السوق. وبالإضافة لهذا الجهد، يجدر ممارسة المسؤولية المشتركة لتقليل المخاطر والتغلب على العوائق وتعظيم الفوائد. ولأن الأمراض المدارية المهملة تقترن اقترانًا وثيقًا بالفقر، فإنه من الضروري للغاية أن يأخذ أصحاب المصلحة تدابير متضافرة وطويلة المدى لمواجهة التحديات المتعددة الأوجه عن طريق تخفيف الفقر المدقع، وتعزيز التدخل الاجتماعي، والتكيف مع التغييرات المناخية، وتزويد مراقبة فاعلة وضمان التسليم في الوقت المناسب.
ستفيد المساعي المستمرة على المستوى العالمي المرضى، والمدن التي يعيشون بها على نحوٍ جوهري، وكلنا أمل أن تُفيد المصنعون الذين يؤمنون منتجات وقائية، وتشخيصية وعلاجية جديدة.

Translated from English version into Arabic by Aseel Abdulrahman and Bashaier Allam, through

TRANSLATORS
WITHOUT BORDERS

被忽视的热带病药物研发的最新进展

Hongbo Weng, Haixia Chen, Ming-Wei Wang

摘要
引言：被忽视的热带病(NTDs)与贫穷密切相关，影响着发展中国家的 10 亿多人。无法接受医治导致了高死亡率和残疾，从而造成巨大的负担，引发严重的社会和经济后果。虽然由世界卫生组织协调，一些慈善组织、国家政府和医药行业一直在努力改善这种状况，但是目前 NTDs 控制力度仍然不够充分，并且极难实施。缺乏安全、有效和价格合理药物是一个关键因素。本文回顾了 NTDs 防控药物研发的最新进展和一些挑战。
主要内容：近年来，许多创新研究已倾向于促进 NTDs 的药物研发。多边协作引导持续的进展，并在药物发现中发挥关键作用。NTDs 药物创新迫切需要积极的方法和先进的技术。但

是，NTDs 控制与消除是一项艰巨的任务，它需要持续的国际合作，才能取得长足可持续发展。目前正在实施之前提出的措施并经验证为有效，其中包括旨在降低产业研发成本的“推”机制，也包括旨在增加市场吸引力的“拉”机制。我们需在全球范围内共同行使责任，以减少风险，克服障碍并实现利益最大化。由于 NTDs 与贫困密切相关，利益相关者必须采取协调一致的长期措施，通过减轻极端贫困，加强社会干预，适应气候变化，提供有效监测和确保及时交付来应对多方面的挑战。
结论：全球范围内的通力协作研发药物将最终惠及患者、他们生活的国家和提供新的预防、诊断和治疗产品的制造商。

Translated from English version into Chinese by Jin Chen, edited by Pin Yang

CHINESE CENTER FOR DISEASE CONTROL AND PREVENTION
NATIONAL INSTITUTE OF PARASITIC DISEASES

Innovation dans la découverte et le développement de médicaments contre les maladies tropicales négligées

Hongbo Weng, Haixia Chen, Ming-Wei Wang

Résumé
Les maladies tropicales négligées (MTN) sont étroitement liées à la pauvreté et touchent plus d’un milliard de personnes dans les pays en développement. Les besoins de traitement à satisfaire sont à l’origine de mortalité et invalidité importantes et, de ce fait, imposent un fardeau énorme aux conséquences sociales et économiques graves. Divers organismes caritatifs, gouvernements nationaux et l'industrie pharmaceutique poursuivent leurs efforts, coordonnés par l’Organisation mondiale de la santé, visant à améliorer la situation ; cependant, à ce jour, le contrôle des MTN demeure inadéquat et extrêmement difficile. Le manque de médicaments sûrs, efficaces et abordables est l’une des principaux facteurs entrant en jeu. Le présent article fait le bilan des récents progrès et de certaines difficultés rencontrées dans la lutte contre les MTN.
Plusieurs innovations de ces dernières années se sont montrées propices à la découverte et au développement de médicaments contre les MTN. La mise en place de collaborations multilatérales engendre des efforts soutenus et joue un rôle crucial dans la découverte de médicaments. La nécessité d'approches proactives et de technologies avancées est urgente dans le domaine de l’innovation pharmaceutique dans la lutte contre les MTN. Il n’en reste pas moins que le contrôle et l’élimination de celles-ci restent une tâche titanesque, puisqu'elles nécessitent une coopération internationale continue afin d'obtenir des progrès durables et prolongés. Quelques stratégies actuelles ont été proposées et se sont avérées fructueuses, qu’il s’agisse de mécanismes de type « push », visant à réduire le coût de la recherche et développement, ou « pull », destinés à accroître l’attractivité des marchés. Cet effort doit se doubler d’un exercice de responsabilité partagée à l’échelle mondiale en vue de réduire les risques, surmonter les obstacles et maximiser les bénéfices. Parce que les MTN sont étroitement associées à la pauvreté, il est indispensable que les parties prenantes engagent des mesures concernées à long terme afin de répondre aux multiples défis qui se présentent en allégeant la pauvreté extrême, en renforçant les interventions sociales, en s’adaptant au changement climatique, en assurant une surveillance efficace et en veillant à la mise à disposition

des traitements en temps utile.
Cette engagement continu à l’échelle mondiale sera finalement bénéfique pour les patients, leurs pays de résidence et, idéalement, les entreprises pharmaceutiques qui fourniront de nouveaux produits prophylactiques, diagnostiques et thérapeutiques contre les MTN.

Translated from English version into French by Suzanne Assenat and Fane Wann, through

TRANSLATORS
WITHOUT BORDERS

Инновации в открытии и разработке лекарств от забытых тропических болезней

Хонгбо Уэнг, Хайшйа Чен, Мин-Уэй Уонг

Аннотация

Забытые тропические болезни (ЗТБ) тесно связаны с бедностью, и более миллиарда человек в развивающихся странах болеют ими. Отсутствие необходимого лечения ведет к высокому уровню смертности и инвалидизации, таким образом, являясь тяжелым бременем с серьезными социальными и экономическими последствиями. Хотя ЗТБ координируются Всемирной организацией здравоохранения, а разные благотворительные организации, правительства и фармацевтические компании прилагают усилия для улучшения этой ситуации, сегодня контроль заболеваемости все еще недостаточен и очень сложно осуществим. Ключевым способствующим фактором является отсутствие безопасных, эффективных и доступных по цене препаратов. В этой статье приведен обзор недавних достижений и некоторых проблем в борьбе с ЗТБ.
В последние годы в ряде инноваций актуализирован интерес к исследованиям и разработке лекарств против ЗТБ. Решающую роль в разработке лекарственных препаратов играет многостороннее сотрудничество на постоянной основе. Для новых лекарств от ЗТБ крайне необходимы прогрессивный подход и передовые технологии. Тем не менее, контроль и искоренение ЗТБ остаётся насущной задачей, требующей постоянного международного взаимодействия для достижения устойчивого прогресса. Некоторые сегодняшние стратегии, которые предложены и проверены временем, включают как механизмы "нажатия", нацеленные на снижение стоимости исследований и внедрения их в производство, так и "вытягивания", нацеленные на усиление рыночной привлекательности. К этому необходимо добавить всеобщую ответственность на глобальном уровне для снижения рисков, преодоления преград и максимизации преимуществ. Так как ЗТБ тесно связаны с бедностью, заинтересованным сторонам абсолютно необходимо принять согласованные и долгосрочные меры для решения разных проблем, среди которых борьба с нищетой, усиление социальной терапии, адаптация климатических изменений, обеспечение эффективного наблюдения и своевременных поставок.
Продолжающиеся изменения на глобальном уровне в конце концов послужат на благо пациентам, странам, в которых они живут, и надеемся, и тем производителям, которые

разрабатывают новые профилактические, диагностические и терапевтические продукты.

Translated from English version into Russian by Dariia Moss and Tatiana Kary, through

TRANSLATORS
WITHOUT BORDERS

Innovación en el descubrimiento y desarrollo de fármacos para enfermedades tropicales desatendidas

Hongbo Weng, Haixia Chen, Ming-Wei Wang

Resumen

Las enfermedades tropicales desatendidas (NTD, por sus siglas en inglés) están estrechamente relacionadas con la pobreza y afectan a más de mil millones de personas en países en vías de desarrollo. Las necesidades de tratamiento no cubiertas son causa de una elevada mortalidad y discapacidad, lo que impone una enorme carga con graves consecuencias sociales y económicas. Aunque coordinadas por la Organización Mundial de la Salud, varias organizaciones filantrópicas, gobiernos nacionales y la industria farmacéutica han estado haciendo esfuerzos para mejorar la situación, el control de las ETD sigue siendo inadecuado y hoy en día es extremadamente difícil. La falta de medicamentos seguros, efectivos y económicos es un factor decisivo. Este documento revisa los avances recientes y algunos de los retos a los que nos enfrentamos en la lucha contra las ETD.

En los últimos años, varias innovaciones han demostrado la propensión a promover el descubrimiento y desarrollo de medicamentos para las ETD. La implementación de colaboraciones multilaterales conduce a esfuerzos continuos y juega un papel crucial en el descubrimiento de fármacos. Se necesitan urgentemente enfoques proactivos y tecnologías avanzadas en la innovación de medicamentos para las ETD. Sin embargo, el control y la eliminación de las ETD continúan siendo una tarea inmensa, debido a que requieren de la cooperación internacional constante para lograr progresos sostenibles durante un largo periodo de tiempo. Se propusieron algunas de las estrategias que ya se utilizan y se corroboró que funcionaban, lo que incluye tanto los mecanismos de «Push», cuyo objetivo es reducir el coste de I+D para la industria, como los de «Pull», cuyo objetivo es aumentar el atractivo del mercado. Este esfuerzo debe ir unido al ejercicio de la responsabilidad compartida a nivel mundial para reducir los riesgos, superar los obstáculos y maximizar los beneficios. Dado que las ETD están estrechamente relacionadas con la pobreza, es absolutamente necesario que las partes interesadas adopten medidas concertadas y a largo plazo para hacer frente a los desafíos polifacéticos gracias a paliar la pobreza extrema, al fortalecimiento de la intervención social, a la adaptación a los cambios climáticos, al suministro de una supervisión eficaz y a la garantía de una ejecución oportuna.

El esfuerzo continuo a escala mundial beneficiará en última instancia a los pacientes, a los países en los que viven y, con suerte, a los fabricantes que suministran nuevos productos preventivos, diagnósticos y terapéuticos.

Translated from English version into Spanish by Guillermo Pinilla Gallego and Syl Figu, through

TRANSLATORS
WITHOUT BORDERS
